# Highway Regional Classification Method Based on Traffic Flow Characteristics for Highway Safety Assessment

**DOI:** 10.3390/s22010086

**Published:** 2021-12-23

**Authors:** Jongdae Baek

**Affiliations:** Department of Highway and Transportation Research, Korea Institute of Civil Engineering and Building Technology (KICT), Goyang 10223, Korea; jdbaek@kict.re.kr; Tel.: +82-31-910-0754

**Keywords:** regional classification, administrative division system, hourly traffic volume ratio, cluster analysis, two-sample goodness-of-fit test

## Abstract

Accurate regional classification of highways is a critical prerequisite to implement a tailored safety assessment. However, there has been inadequate research on objective classification considering traffic flow characteristics for highway safety assessment purposes. We propose an objective and easily applicable classification method that considers the administrative divisions of South Korea. We evaluated the feasibility of this method through various theoretical analysis techniques using the data collected from 536 permanent traffic volume counting stations for the national highways in South Korea in 2019. The ratio of the annual average hourly traffic volume to the annual average daily traffic was used as the explanatory variable. The corresponding results of factor and cluster analyses with this ratio showed a 61% concordance with the urban, suburban, and rural areas classified by the administrative divisions. The results of two-sample goodness-of-fit tests also confirmed that the difference in the three distributions of hourly volume ratios was statistically significant. The results of this study can help enhance highway safety and facilitate the development and application of more appropriate highway safety assessment tools, such as Road Assessment Programs or crash prediction models, for specific regions using the proposed method.

## 1. Introduction

When considering approaches for the maintenance and improvement of human health, regular checkups to monitor and precautions for possible health problems are important in addition to lifestyle changes such as healthy eating habits and exercising. Highways, which are an essential social overhead capital, are frequently compared to human blood vessels in their functions. Road crashes can incur loss and damage to human life and property, and this can adversely affect the overall health and well-being of human society. Thus, the appropriate diagnosis and assessment of highway safety is a critical task that must be performed to save lives by preventing accidents through the planning and implementation of an efficient highway safety improvement program. According to the Global status report on road safety 2018, which was released by the World Health Organization (WHO) in December 2018, approximately 1.3 million people die every year because of road traffic crashes worldwide [1]. As of 2018, South Korea witnessed 1.4 road crash deaths per 10,000 vehicles, which caused it to rank thirty-first among the 36 OECD member countries. For 2020, there were 209,654 cases of road crashes, which led to the deaths of 3081 people [2]. These figures suggest that road safety is a matter of high priority in our society.

Among the many different methods for highway safety assessment, the most common method is to estimate the likelihood of crashes based on road factors. That is, the findings obtained by analyzing the correlation between road crashes that occurred in the past and environmental factors such as the road condition at the time of the crash and the characteristics of traffic flow can be developed into assessment tools such as assessment indicators or models and applied for highway safety assessment. Representative examples of these methods include the Highway Safety Manual (HSM) established using the crash prediction model [3] and the Road Assessment Program (RAP) that assesses road safety for classification based on the road environment [4].

Crash prediction models are statistical models used for representing the relationship between variables using statistical techniques such as regression analysis with road environmental factors that affect road crashes as independent variables and crash frequency as a dependent variable. Here, the relationship between the independent and dependent variables is considerably affected by the geographical location of the road, and it is therefore important to develop and apply a model tailored to the regional characteristics. For example, the annual average daily traffic (AADT) is included as an essential independent variable in the crash prediction model because the likelihood of crashes is most strongly associated with traffic volume. However, even with the same AADT, the traffic volume may be differently distributed according to the time of a day as per the region where the road is located; further, it may affect the likelihood of crashes. Therefore, the traffic flow characteristics can be incorporated into the model by developing a crash prediction model through classifying into regional types. This method allows the effect of complex actions of individual factors that cannot be fully considered in the model as variables to be reflected. In addition, the HSM advises establishing a separate crash prediction model by differentiating between urban and rural areas [3]. This case is comparable to the need to apply various assessment indicators to determine the level of health in the health checkup by developing indicators differentiated by gender and age group. Therefore, it is necessary to group these roads with similar traffic flow characteristics, classify them into several types, and develop an assessment model suitable for the type for application.

The regional classification of highways is considered an important prerequisite to implement a tailored safety assessment that is based on the traffic flow characteristics of respective highways. The method of the regional classification of highways differs according to the purpose of the classification. For safety assessment purposes, highways are mostly classified into two or three types according to the characteristics of the region where they are located. For example, a region is classified into three areas: urban area, rural area, and the suburban area as the area between the two, in the HSM. However, there are very few studies on objective methods or criteria that focus on the regional classification of highways such as quantitative reference values for highway safety assessment. Thus, considerable research is required to develop objective indicators for the regional classification of highways.

Further, the classification method should be easily applicable and should be based on objective indicators. This is because a considerable amount of time and resources can be saved in the highway safety assessment if efforts are not invested in collecting and analyzing a large amount of data such as the level of urbanization and socio-economic activities in the region.

This study proposes a method for utilizing the administrative division system for the easy and objective regional classification of national highways among various types of roads in South Korea. For this purpose, we evaluated the adequacy of the proposed method using theoretical analysis techniques. The administrative divisions serve not only as spatial units for region-specific administration, but also as living spaces where a regional identity can be created [5]. Human life patterns are affected by spatial characteristics, and road traffic patterns can be considered to be related to these life patterns.

The proposed method has distinct advantages in that it accurately classifies regional characteristics and minimizes the time and effort required for data collection. The results of this study can improve the regional classification of highways and contribute to enhancing highway safety by facilitating the development and application of more appropriate assessment models. For example, for the RAP, the proposed method can facilitate the implementation of more accurate assessments by applying the road protection score (RPS) suitable for each region considered for each assessment item used for the rating. Further, in highway safety assessment, especially for the national highway system, the distinction between suburban and rural areas is very important in that the factors affecting road safety and the magnitude of their impact vary depending on the region. However, there is no quantitative and objective method to distinguish suburban areas from rural areas.

The rest of this manuscript is organized as follows: The review of related literature is provided in Section 2, followed by the design of the analysis method in Section 3, and the analysis and results in Section 4. Finally, the conclusions are drawn in Section 5.

## 2. Literature Review

May [6] presented in his book that traffic flow rates vary over time, and different types of highway facilities have different traffic flow variations. He also said that traffic flow rates vary by area types. He presented figures showing hourly flow patterns on the San Francisco-Oakland Bay Bridge and a typical intercity route as examples to show the differences in traffic flow characteristics in urban and rural areas. These figures showed that urban highways had two peak flows during rush hours and rural highways had single peak flow periods during the late afternoon. He merely explained the macroscopic flow characteristics and temporal flow patterns, especially daily flow patterns varying by area types; however, he did not present a detailed method to classify highway regional types using those traffic characteristics.

For the classification of road types, Albright [7] used a cluster analysis method for a more accurate conversion of short-term traffic counts into average daily traffic values. His research converted permanent traffic counter stations in New Mexico, USA, into groups with similar monthly traffic factors. This study, however, classified roads based on traffic volume characteristics and not as the regional classification of roads. Flaherty [8] conducted a similar study a few years later. In his research, cluster analysis was performed using monthly factor data based on five years of data collected from 28 permanent traffic volume counting stations installed in Arizona, USA. His research aimed to classify 28 stations into groups with similar monthly variation patterns in order to obtain the means of the monthly factors of each group, which were required to estimate the annual average daily traffic (AADT) using short-period traffic counts.

Zambon et al. [9] grouped the roads in Milan city into two groups having similar noise patterns to obtain noise maps of the city as part of a project named dynamic acoustic mapping (DYNAMAP). They conducted a cluster analysis using noise levels to classify urban roads with similar noise trends. They applied this statistical approach because they needed something better than the legislative road classification to reflect the actual use of roads and, thus, the actual noise emissions. This study, however, classified roads based on the temporal behavior of noise from roads, allowing for a more realistic description of road networks but not for the regional classification of roads taking into account traffic flow characteristics.

The need for classifying highways based on geographic regions is specified in the HSM [3]. The HSM is a guidebook that enables the inclusion of scientific and numerical safety analysis in the planning and development process of highway transportation projects. To this end, the HSM proposes a method for estimating the expected average crash frequency of the target highway using safety performance functions (SPFs) and crash modification factors (CMFs). The HSM suggests developing and applying the SPF and CMF differently based on the region where the highway is located. The regions are classified into urban, suburban, and rural areas; as criteria for the classification, the HSM proposes considering roadway characteristics, surrounding population, and land use. However, the HSM does not propose clear quantitative criteria for classification, and it states that decisions should be made based on the discretion of the users. In the HSM, a suburban area is defined as the area outside the urban area; however, in terms of the crash prediction method, there is no difference between the urban and suburban areas.

The Highway Capacity Manual (HCM) [10] suggests that the K-factor, the proportion of AADT occurring in the analysis hour, should be determined from local data for similar facilities with similar demand characteristics. HCM presented an example of K-factors by area types developed for Florida. The Florida Department of Transportation classified the area types as urbanized, urban, transitioning, rural developed, and rural undeveloped. Urban areas are defined as areas with a population of at least 5000. Transitioning areas are the areas outside of, or urbanized areas expected to be included in, an urbanized area within 20 years. However, this classification criterion is for the classification of roads in terms of road design standards and does not consider traffic flow characteristics crucial for safety assessment. Furthermore, this criterion is qualitative; not only does it fail to present numerical criteria, but it also takes considerable time and effort to collect the data necessary for use.

In South Korea, studies were conducted to classify the types of national expressways and highways; however, these studies aimed at the functional classification of national highways, classification to present design standards for roads, and classification for discrimination of recreational roads [11,12,13,14,15,16,17,18,19,20]. Therefore, these classifications were not intended for highway safety assessment. Oh et al. [13] conducted factor and cluster analyses using various road traffic indicators for the regional classification of highways targeting national highways; they divided highways into four groups: urban roads, urban/rural roads, rural roads, and recreational roads. Choi et al. [20] classified the regional types of freeways through cluster analyses using the annual variance of hourly traffic volume on freeways. They found that the coefficient of variance of freeways in urban areas is lower than that of rural areas. As a result, they classified the freeways into five groups: freeways in metropolitan, urban, rural, rural-recreational, and recreational areas. However, their study aimed at analyzing the functional classification of roads considering road planning or road design. Further, nearly 20 years old data were used in that study, and therefore, there is a need to conduct a reanalysis based on more recent data considering the fast-paced changes occurring in South Korea.

**Figure 1:**
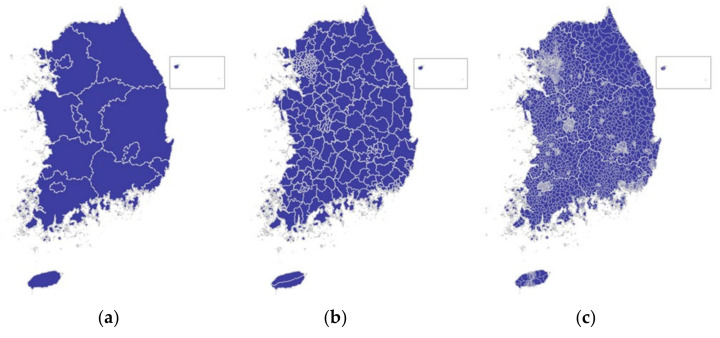
Administrative divisions of South Korea [5]: (**a**) Principal level: special city, metropolitan city, province; (**b**) municipal level: city, county, and district; (**c**) sub-municipal level: town, township, and neighborhood.

As shown in Figure 1, administrative divisions of South Korea comprise 17 principal-level local governments (special cities, metropolitan cities, and provinces) and 226 municipal-level divisions (cities, districts, and counties). The cities and districts tend to be more urbanized areas and the counties are rural areas. These areas are further divided into a total of 3,498 sub municipal level regions [21]. Areas at the sub municipal level are divided into three types: areas ending in -eup (corresponding to a town) in Korean names, areas ending in -myeon (corresponding to a township), and areas ending in -dong (corresponding to a neighborhood). A dong is the smallest unit administrative division of urban governments such as a city or district; eup and myeon are administrative divisions on the outskirts of the city area excluding dongs or in the county. Therefore, these regions have fewer urban characteristics compared to those of dongs.

According to the Local Autonomy Act in Korea, there are several eligibility requirements to classify as a city. In most cases, “a region in an urban form with a population of at least 50,000” is used as the classification criteria [22]. Areas that are not classified as urban areas are classified as rural areas because there are no legal requirements for classifying rural areas. In the Rules on the Structure and Facilities of Roads, which is a road-related legislation, an urban area is one that forms a built-up area or an area likely to be formed into a built-up area; a rural area is defined as an area besides the urban area [23]. Thus, there is no objective criteria for the classification of regions. In the Korea Transport DataBase (KTDB), a dong is specified as an urban area and eup and myeon are specified as rural areas in terms of administrative division [24].

South Korea has had an unprecedented level of rapid economic growth. There has been an increased focus on the function of national highways, which form the national arterial road network with national expressways, as arterial roads given the rapid pace of economic growth and the expansion of the road network infrastructure. Thus, highways are being actively constructed as detour routes that bypass the existing section for those that pass through established city areas (e.g., those with considerable urbanized development); this has led to a decrease in national highways that are part of urban areas. With an increase in the number of urban areas, regions that exhibit characteristics of rural areas near the urban area gradually acquires the characteristics of an urban area. Thus, there is an increased need to classify the regional types of highways not only into urban area and rural area, but also in the suburban area.

The literature review described above suggests that regional classification is necessary to perform highway safety assessment more accurately and effectively; in this regard, there exists a need for an objective and highly applicable method for the regional classification of highways. Further, it shows that it is possible to examine a regional classification method for highways based on administrative divisions because the administrative divisions of South Korea are determined based on living zones with similar life patterns, which indicate a similarity in traffic patterns. Furthermore, it shows a need for using latest data considering the dynamic pace of development in South Korea.

## 3. Design of the Analysis Method

This study aims to develop a highly applicable method for the classification of national highways into several groups with similar regional characteristics for increasing the accuracy of the safety assessments of national highways in South Korea. This method classifies highways into several groups based on similar traffic flow characteristics that have a significant impact on highway safety; accordingly, tailored safety assessment indicators for each classified group can be developed. For this highway classification, the accuracy and applicability, i.e., the ease of data acquisition and analysis, are important parameters. This study proposes using the administrative division system as classification criteria, and it evaluates the adequacy of the proposed method based on theoretical analysis techniques. The administrative division system was considered in this study because administrative regions are divided based on a consideration of living zones with similar land use characteristics and life patterns; the traffic flow characteristics are closely related to these characteristics of living zones.

**Figure 2:**
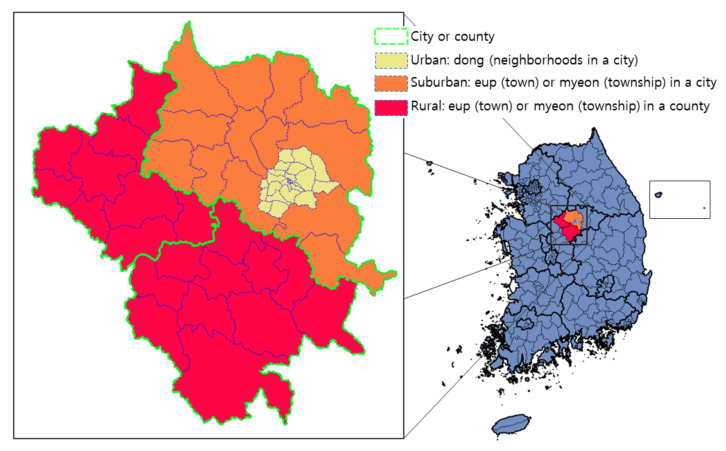
Concept of classifying an area as urban, suburban, or rural using sub-municipal administrative divisions in South Korea (created using the shapefile from [5]).

In this study, the regional classification for highway safety assessment was divided into urban, suburban, and rural areas based on the fact that the characteristics of the living zones in these areas are well distinguished. This study attempts to suggest a method for the classification of highways into one of those regions using sub-municipal level administrative divisions (e.g., eup, myeon, and dong). As shown in Figure 2, the dong area, which is the area with the highest urbanization within a city, is classified as the urban area; eup and myeon located within the city boundary and around the dong are classified as suburban areas; and other eup and myeon belonging to counties are classified as rural areas. In the data of South Korea used for the analysis of global urbanization prospects by the UN, areas defined as dong in the administrative division are set as urban areas [25].

Two theoretical analysis techniques were used to evaluate the adequacy of the proposed method. One method uses the traffic flow characteristics of highways as indicators and performs the regional classification by applying factor and cluster analyses; the results were compared with the classification based on the administrative division system. The other method examined whether the three groups classified by the administrative division system are different groups with differentiated characteristics using a two-sample goodness-of-fit test method. In this method, the traffic flow characteristics of highways were also used for the test. The analysis was performed on the national highways of South Korea. For the data, the traffic volume data collected from 536 permanent traffic volume counting stations in 2019 on the national highways were used to analyze the traffic flow patterns of national highways in each region. Further, an hourly traffic volume ratio to a 24-h traffic volume—an important factor for road safety analysis—was selected among various traffic flow characteristics that can be identified through the traffic volume data.

Factor analysis uses correlation information (e.g., covariance and correlation) between observed variables to group closely related variables, i.e., variables with similar attributes. The method is used to reduce the number of variables used in the analysis or research so that the research or analysis can be performed with only selected key factors [26]. This method has the advantage of reducing the redundancy between a number of variables [27]. Further, a factor analysis is employed for the brief and accurate encapsulation of correlations between directly observed variables for aiding the conceptualization of certain facts or theories that need to be identified [28]. In this study, the 24 annual average hourly traffic volume ratios to AADT were used as the explanatory variables. These 24 variables were categorized into several groups (e.g., factors) by grouping them with highly correlated variables using factor analyses; the reduced number of factors were used for the cluster analysis.

The cluster analysis technique is one wherein the given data are grouped into several clusters based on similarities in terms of certain attributes to analyze the characteristics of each cluster. This technique is divided into hierarchical cluster analysis and nonhierarchical cluster analysis. Nonhierarchical cluster analysis, which assigns measurements to clusters, is appropriate when the number of clusters is predetermined by the researcher. In this study, the factors derived through the factor analysis were used, and with the non-hierarchical cluster analysis method, 536 permanent traffic volume counting stations were classified into three clusters; the results were compared with those obtained from the classification by administrative divisions.

**Figure 3:**
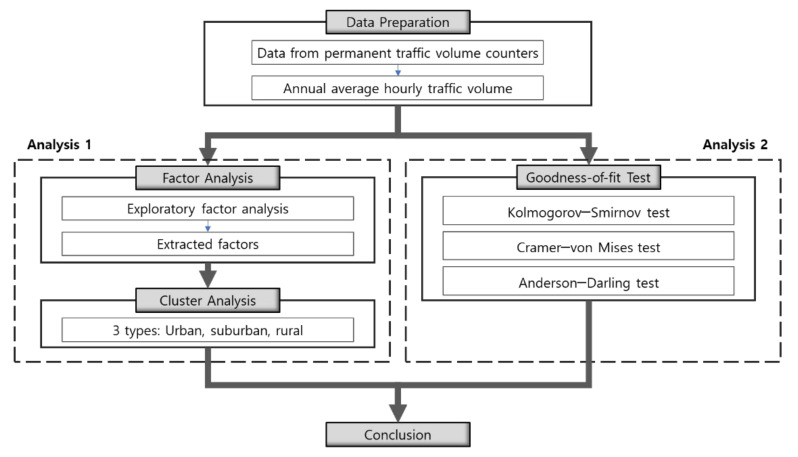
Flowchart of the analysis process.

The two-sample goodness-of-fit test technique verifies whether the distribution of the two data samples has no significant difference between them. There are several methods for implementing this technique; the most well-known and widely used technique is the Kolmogorov–Smirnov (KS) test proposed in 1939 by Smirnoff [29]. Other representative methods include a Cramer–von Mises (CVM) test [30,31], Anderson–Darling (AD) test [32], and Mann–Whitney (MW) test [33]. For each method, the power of the test varies depending on the scenario [34]. Further, there is a report that the AD test achieves better power than the KS test [35]. Therefore, in this study, in addition to the KS test, the CVM test—reported to be effective in testing the difference in the location of the sample or scale—and the AD test—reported to be effective in testing the difference in the degree of bias or spread of the sample—are additionally performed. RStudio (version 4.0.5) [36] was used for all statistical analyses. The flow of the analysis process employed in this study is illustrated in Figure 3.

## 4. Analysis and Results

### 4.1. Data Collection

The national highways of South Korea connect major cities, designated ports, major airports, national industrial complexes, or tourist destinations to form the national arterial road network with the national expressways. The routes of national highways are designated and notified by the Minister of Land, Infrastructure and Transport [37]. At the end of 2019, the total length of national highways was 14,030 km [38]. Automatic vehicle classification (AVC), which is a monitoring system that can count and classify traffic volume and vehicle type simultaneously, is installed for monitoring traffic volume in terms of vehicle type, direction, and hour of the day, 24/7 for 365 days, to perform the permanent traffic volume counting of national highways [39]. In this study, data from a total of 536 permanent traffic volume counting stations installed on national highways were collected and used for the analysis. Although the data for 2020 are the latest available data, data for 2019 were used instead because there could be changes in traffic flow behavior in 2020 which could be attributed to the COVID-19 pandemic.

The hourly traffic volume ratio was analyzed for traffic volume during weekdays (Monday to Friday) excluding public holidays using the data collected for one year. The hourly traffic volume ratio refers to the ratio of hourly traffic volume to the 24-h traffic volume. For each permanent traffic volume counting station, the annual average hourly traffic (AAHT) is obtained for each hour of the day, and it is divided by the AADT of the station to obtain the annual average hourly traffic volume ratio to AADT; this ratio is used for the analysis. The annual average hourly traffic volume ratio to AADT is calculated as:

(1) $$H_{i}=\frac{AAHT_{i}}{AADT}=\frac{AAHT_{i}}{\sum_{i=1}^{24}AAHT_{i}}$$

where *Hi* denotes the annual average hourly traffic volume ratio to AADT for hour *i*, and *AAHTi* denotes the annual average hourly traffic volume for hour *i*.

**Figure 4:**
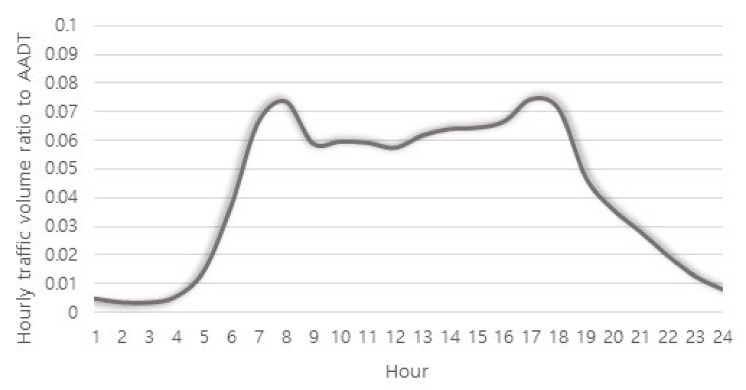
Annual average hourly traffic volume ratio to AADT (weekdays) for the national highways in Korea.

Figure 4 shows the distribution of the annual average hourly traffic volume ratio to AADT for all 536 stations as a graph. A typical two-humped shape is observed, and it well represents the concentration of traffic during the peak hours in the morning and afternoon on a typical day. In this study, H_1_ indicates from the period from 00:00 to 01:00, H_2_ from 01:00 to 02:00, and H_24_ indicates from 23:00 to 24:00.

### 4.2. Factor Analysis

Factor analysis was performed to determine the grouping of 24 annual average hourly traffic volume ratio to AADT variables. The exploratory factor analysis (EFA) method was used. The principal component method was used to minimize the loss of information while minimizing the number of factors when extracting the factors.

First, the Bartlett sphericity test was performed to determine whether there is sufficient correlation between variables and to determine the adequacy of the factor analysis. The resulting *p*-value was *p* = 0.000 < 0.05, and therefore, the null hypothesis that the correlation coefficient matrix between variables was the identity matrix was rejected, which implies the presence of the correlation between the variables. Therefore, it was confirmed that the factor analysis can be performed.

Next, eigenvalues were analyzed to determine the number of factors, and the eigenvalue is a measure of how much the variance of the observed variables can be explained by a factor. Any factor with an eigenvalue ≥1 indicates that the factor explains the variance of at least a single observed variable [40]. Therefore, there are four factors with the eigen value ≥1 as indicated in Table 1; these factors are selected as the main factors to perform the rest of the factor analysis.

The matrix was rotated using the Varimax method to transform the factor matrix representing the correlation between the factors extracted above and the variables into a form for easy interpretation. Table 2 summarizes the rotated factor matrix, the eigenvalues, and the cumulative variance of each factor. The analysis indicated that the four factors explain 82.2% of the total variance.

The criterion used to assess the convergent validity for examining whether the variables reflected in the same factor have high correlation is based on fact that the absolute value of the factor loading value is above a certain value. The required minimum factor loading value varies based on the size of the sample; the required minimum factor loading value is 0.3 because the sample size in this study is over 350 [41]. The larger the factor loading value of a variable for a certain factor, the greater is the degree to which the factor explains the variable. In Table 2, factor loading values with absolute values ≥0.5 are indicated in bold.

**Figure 5:**
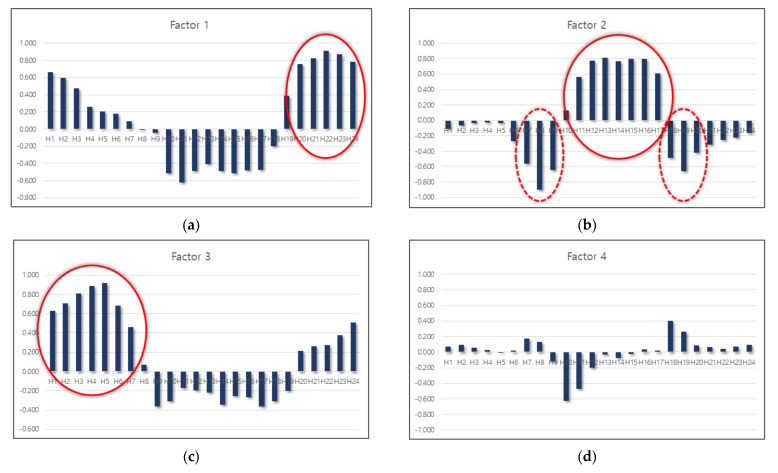
Hours with high factor loadings: (**a**) Factor 1; (**b**) factor 2; (**c**) factor 3; (**d**) factor 4.

It is possible to determine the variable mainly explained by a factor based on the factor loading values presented in the factor matrix in Table 2. Thus, it is possible to name factors based on their characteristics [42]. To this end, a column chart is prepared as shown in Figure 5 for the easy visualization of the magnitude and sign of factor loading values of each factor. As shown in Figure 5, the column size of the variables explained by the factor appears to be longer (up or down), which makes it easier to identify the characteristics.

The most pronounced characteristic is that, for each factor, variables with a high correlation are concentrated in a specific part (encircled part in Figure 5). In factor 1, the factor loading value is large at hours 20–24; in factor 2, at hours 07–09, 11–17, and 18–20; and in factor 3, hours 1–6. In factor 4, there was only one variable with a factor loading value >0.5; thus, the factor loading values of the variables other than this were small and did not show a clear pattern, which indicates that their characteristics were not well presented. The eigenvalue of factor 4 is 1.009, which is considerably smaller than that of the previous factors, and this is believed to be relevant to the difference.

**Figure 6:**
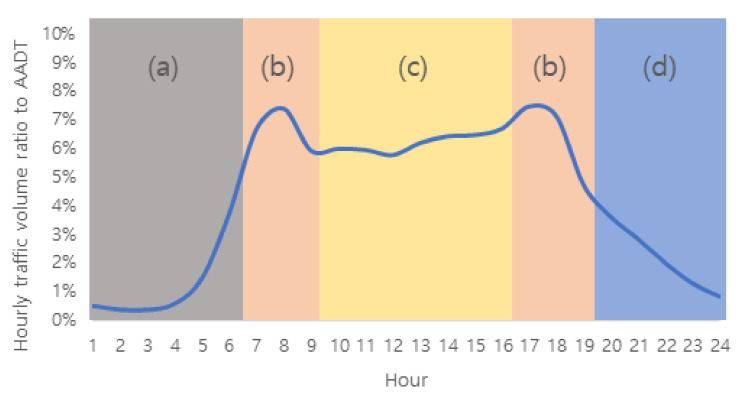
Hour groups extracted from the factor analysis: (**a**) Early morning hours; (**b**) peak hours in the morning and afternoon; (**c**) day off-peak hours; (**d**) night hours.

In the factor analysis, the night and early morning hours are separately classified as in factors 1 and 3; the daytime hours are grouped into one as shown in factor 2. As indicated in Figure 4, the characteristic that the traffic volume of daytime hours is significantly higher than other hours is explained. However, Figure 5b shows that it is necessary to consider the signs as well as values of the major variables explained by factor 2. There is a clear differentiation between the group with a positive sign (a solid circle) and a group with a negative sign (a dotted line circle); further, the groups match with day off-peak hours and peak hours in the morning and afternoon, respectively. Thus, based on the interpretation of the factor analysis results, it was determined appropriate to group the 24 annual average hourly traffic volume ratios to AADT variables into four hour zones (Figure 6) as follows:

1. H01–H06: Early morning hours;
2. H07–H09 and H18–H19: Peak hours in the morning and afternoon;
3. H10–H17: Day off-peak hours; and
4. H20–H24: Night hours.

### 4.3. Reliability Analysis of the Annual Average Hourly Traffic Volume Ratios to AADT Groups

According to the result of factor analysis, the 24 h were grouped into separate hour zones assessed to be highly correlated and classified into four hour groups. A reliability analysis was performed to evaluate whether this classification approach was valid. For the reliability analysis, the Cronbach’s alpha value method—the most well-known and commonly used method—was used. The analysis results are outlined in Table 3. For all hour groups, the Cronbach’s alpha values were ≥0.7, which confirm that the grouping method of variables is highly reliable.

### 4.4. Cluster Analysis

Cluster analysis was performed wherein permanent traffic volume counting stations of national highways located across the entire land of South Korea were grouped into three clusters using the annual average hourly traffic volume ratio to AADT for each of the four hour groups extracted through the factor analysis as a characteristic variable. This was done to evaluate the extent to which the three clusters classified through the cluster analysis match with the regional classification performed using the administrative division system.

To this end, the average value of the annual average hourly traffic volume ratio to AADT of the hours belonging to each of the four hour groups derived above was obtained for each permanent traffic volume counting station; this value was set as the representative annual average hourly traffic volume ratio to the AADT value of the hour group. Cluster analysis was performed using four methods of k-mean clustering (Hartigan-Wong, Lloyd, Forgy, and MacQueen), which is a non-hierarchical cluster analysis technique, using this value as a characteristic variable, and the fitness was compared with the BetweenSS/TotSS values.

Different scenarios were composed by encompassing the case of using all four characteristic variables determined in the factor analysis and those obtained using the selected characteristic variables among the four characteristic variables because the fitness of the cluster analysis results depend on the number of characteristic variables. Further, cluster analysis was performed using the scenarios outlined in Table 4.

The number of stations for each area is 35, 205, and 295, respectively, when 536 permanent traffic volume counting stations are classified into urban, suburban, and rural areas based on the administrative division system; this implies that the number of stations that belong to the urban area is considerably smaller than that of the other two. Since this study was conducted on national highways, the number of national highways belonging to the urban area is small, and thus, the number of stations in the urban area is small. This study aimed to compare the results of cluster analysis using traffic flow characteristics with classification using the administrative division system.

However, no attempt was made in the cluster analysis to ensure the number of individuals in each type when the number of individuals belonging to each type on classification established by the administrative division system showed differences as large as described above. Therefore, it may not be appropriate to compare the classification results of the two methods in terms of similarity. To address this problem, only 35 stations belonging to the suburban and rural areas were randomly selected because there are 35 stations in the urban area. Thus, the number of individuals for each type of the region is the same, and the sub-data constructed based on this number of stations to perform the analysis were 105 in total.

Table 5 summarizes the results of the cluster analysis for each scenario. The values of BetweenSS/TotSS did not show a significant difference based on the four methods; the values were arranged in the ascending order based on the Hartigan–Wong value. The smaller the number of variables used in the analysis, the larger was the BetweenSS/TotSS value. If these values are greater than 0.6, the adequacy of the cluster analysis result is considered to be good. The results of the cluster analysis obtained using 13 out of 15 scenarios indicated that BetweenSS/TotSS ≥ 0.6, which implies the adequacy of the method. Scenario 14, which showed the highest fitness value, used the day off-peak hours as a variable. It can be interpreted that the day off-peak hours suggest the hourly traffic volume ratio characteristic that can be best classified by the three clusters.

One point was given for the top 5 scenarios with high fitness from the cluster analysis (scenarios 14, 15, 12, 7, and 8), when the three types of region classified in the cluster analysis and the administrative division was the same; if not, 0 point was given for 105 stations used in the cluster analysis to evaluate the similarity between the classification results using cluster analysis and the classification using the administrative division system. For each scenario, the total score calculated by adding up the scores of all 105 stations was divided into 105; this value was used as an index to determine the fitness in percentage. Table 6 summarizes the fitness score for each scenario. The highest fitness score is 61%, which indicates that the regional classification using the administrative division system and the classification performed using the cluster analysis based on the traffic flow characteristics showed an agreement of 61%.

In this study, only one explanatory variable—the annual average hourly traffic volume ratio to AADT—was used for the factor and cluster analyses. There are many other traffic flow characteristics that can be considered for the regional classification of highways, including peak-hour ratio, heavy vehicle proportion, day-night volume proportion, and variation in the day of the week (weekend characteristics), besides the annual average hourly traffic volume ratio to AADT. The result of cluster analysis showed fitness over 60% with the classification using the administrative divisions, which is a significant finding obtained using only the variable of annual average hourly traffic volume ratios to AADT.

**Figure 7:**
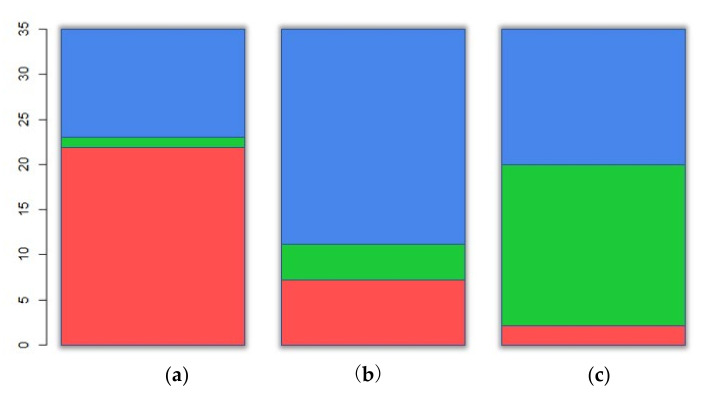
Result of the clustering analysis for scenario 8 in number of stations of each cluster: (**a**) rural; (**b**) suburban; (**c**) urban.

**Figure 8:**
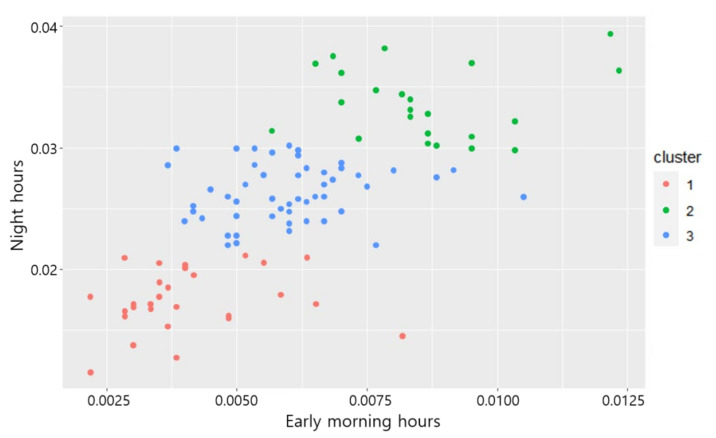
Scatter plot of the result of the clustering analysis for scenario 8.

The variable used in scenarios Nos. 8 and 12, which showed 61% fitness, is early morning hours. Thus, the variable that showed the highest level of agreement between the classification by cluster analysis and the regional classification of highways using the administrative division system is the annual average hourly traffic volume ratio to AADT in early morning hours. Figure 7 and Figure 8 illustrate the results of cluster analysis based on scenario 8 as a graph.

### 4.5. Two-Sample Goodness-of-Fit Tests

In this study, the goodness-of-fit was evaluated for the annual average hourly traffic volume ratio to AADT distribution in regions classified as urban, suburban, and rural areas by the administrative division system. As explained previously, the KS, CVM, and AD tests were used for the evaluation.

First, 536 permanent traffic volume counting stations were divided into urban, suburban, and rural area groups with the classification method using an administrative division system to prepare data for the analysis as done in the cluster analysis. Next, the average value of the annual average hourly traffic volume ratio to AADT of the stations classified into each group is used and the distribution of the annual average hourly traffic volume ratio to AADT of each of these groups is calculated. For each group, Table 7 outlines the annual average hourly traffic volume, annual average hourly traffic volume ratio to AADT, and cumulative annual average hourly traffic volume ratio to AADT to be used as the empirical cumulative distribution function (ECDF) in the analysis.

**Figure 9:**
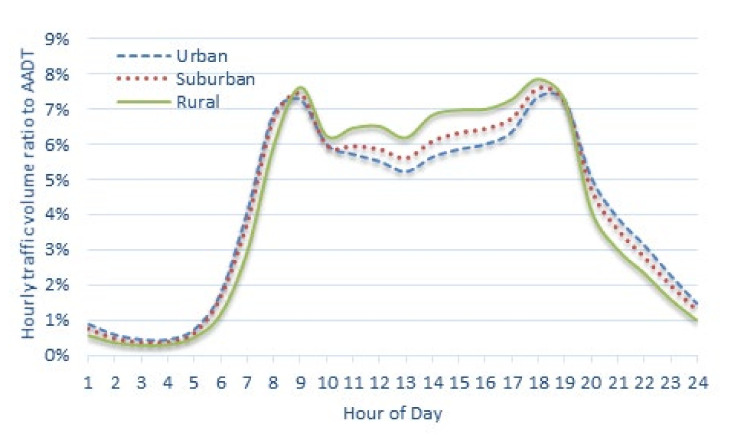
Annual average hourly traffic volume ratios to AADT (weekdays) of national highways of the urban, suburban, and rural areas (nationwide).

Figure 9 shows the annual average hourly traffic volume ratio to AADT for each type of region presented in Table 7 as a graph. In Figure 9, the difference in the distribution of each region type can be confirmed visually. In the urban area, a two-humped shape observed where a considerable amount of traffic is concentrated during the peak hours of commuting to and from work is dominant, whereas in the rural area, this type of peaks is less pronounced. The suburban area shows an intermediate trend between the urban and rural areas.

Two-sample goodness-of-fit test techniques—KS, CVM, and AD tests—were performed using the values presented in Table 7 to examine whether the difference in the distribution of the three regional type is statistically significant. The results of the tests are presented in Table 8. In all three tests, it was determined that the difference in the distribution of the three regional type was statistically significant since the *p*-value was less than 0.05.

**Figure 10:**
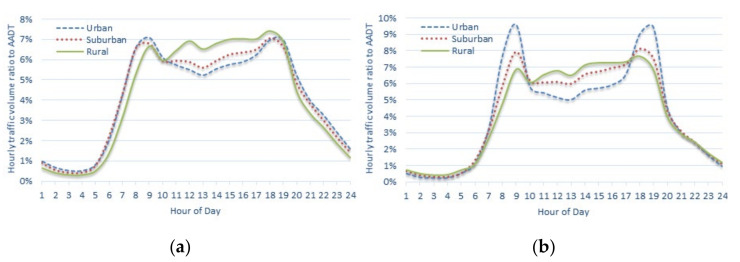
Annual average hourly traffic volume ratios to AADT (weekdays) of national highways of the urban, suburban, and rural areas: (**a**) Gyeonggi-do; (**b**) Gangwon-do.

Further, the test was also conducted at units of principal level administrative division rather than that at the national unit. Among the 17 principal level divisions shown in Figure 1a, there are seven administrative divisions with permanent traffic volume counting stations located in all urban, suburban, and rural areas. Among these, the analysis was performed on Gyeonggi-do, which is the most urbanized province, and Gangwon-do, the province with the strong rural area characteristics attributed to the presence of numerous mountainous areas. The results are shown in Figure 10 and Table 9.

As indicated by the analysis results of the nationwide units, the difference in the distribution of the three regional types can be visually confirmed in Figure 10 for both Gyeonggi-do and Gangwon-do. The characteristics of the urban, suburban, and rural areas show a clear differentiation in Gangwon-do, which has fewer urbanized areas than in Gyeonggi-do with more urbanized areas. As indicated in Table 9, the statistical tests confirmed that the distribution of traffic flow characteristics in each regional type was not identical.

In conclusion, the results confirm that the proposed method is appropriate for regional classification because there are statistically significant differences in the distribution of traffic flow characteristics in the urban, suburban, and rural areas, which were classified using the administrative division system.

## 5. Conclusions

The appropriate regional classification of highways is a critical prerequisite to implement tailored safety assessment. This is because most highway safety assessment methods estimate the likelihood of crashes based on road factors such as traffic flow characteristics that are considerably affected by the geographical location of respective highways. This is comparable to the need to develop and apply differentiated health checkup indicators by gender and age group in health checkups. However, the criteria for the regional classification of highways differ by country, and furthermore, there are very few studies on objective criteria that focus on the regional classification of highways, such as quantitative reference values for highway safety assessment.

In this study, an objective and easily applicable method for classification of the regional types of highways using the administrative division system was proposed. The feasibility evaluation of this method through statistical analyses indicated that the administrative divisions effectively distinguish the traffic flow patterns by region. Moreover, this result was obtained by applying only one explanatory variable of annual average hourly traffic volume ratio to AADT; an improved result can be expected with the application of other variables that well represent the traffic flow characteristics in the future. For these reasons, the method proposed in this study is highly significant.

The most distinctive advantage of the proposed method is that it allows to minimize the time and efforts required for data collection required for applying the method and realizes the proper classification of regional characteristics. The results of this study can be used to develop and apply more suitable highway safety assessment tools that can help classify highway regional types easily and conveniently using the proposed method. This can help improve highway safety. For example, in the RAP, a more accurate assessment can be achieved by varying the RPS for each assessment item used for the star rating for each region. Among the assessment items, the level of safety according to the speed is assessed through RPS, and a speed of 60 km/h may have a different effect on safety based on whether the highway is in an urban or a rural area.

The proposed method, however, has limitations in some cases in that the traffic flow characteristics of an area can be closer to those of the suburban area when the area is located closer to the urban area but classified as rural area on the administrative division; however, this cannot be reflected in the proposed method. There could also be opposite cases. That is, although an area is classified as a suburban area in the administrative division, there may be cases where it is located far from the city center and has traffic flow characteristics closer to those of a rural area.

Therefore, further research to establish improved classification criteria, combining the traffic flow characteristics with the administrative divisions, would be needed in the future. For example, if the roads first classified using the method proposed in this study were classified again using the traffic flow characteristics, such as annual average hourly traffic volume ratio to AADT, the accuracy of the regional classification of roads could be further improved. It would also be worthwhile to conduct a further study on the suitability of the method of regional classification based on the distance from the urban area, that is, the regional classification method using concentric circles at some distance from the center of the city. Efforts to develop quantitative classification criteria for these methods should also be made. Highway regional classification using AI or deep neural network methods should also be studied in the future to enhance the accuracy and applicability.

## Figures and Tables

**Table 1:** Results of the eigenvalue analysis.

Factor 1	Factor 2	Factor 3	Factor 4	Factor 5	Factor 6	Factor 7	Factor 8
13.674	4.290	1.731	1.075	0.899	0.421	0.374	0.291
Factor 9	Factor 10	Factor 11	Factor 12	Factor 13	Factor 14	Factor 15	Factor 16
0.214	0.150	0.146	0.127	0.107	0.092	0.080	0.076
Factor 17	Factor 18	Factor 19	Factor 20	Factor 21	Factor 22	Factor 23	Factor 24
0.063	0.050	0.039	0.038	0.028	0.019	0.016	0.002

**Table 2:** Factor loading matrix (Varimax rotation), eigenvalues, and cumulative variance (CV).

Factor	Factor 1	Factor 2	Factor 3	Factor 4
H1	0.666	−0.114	0.628	0.073
H2	0.595	−0.064	0.707	0.093
H3	0.477	−0.038	0.806	0.057
H4	0.260	−0.030	0.889	0.028
H5	0.203	−0.037	0.916	−0.003
H6	0.179	−0.265	0.682	0.018
H7	0.091	−0.558	0.459	0.178
H8	−0.011	−0.897	0.072	0.129
H9	−0.043	−0.648	−0.364	−0.122
H10	−0.517	0.126	−0.310	−0.629
H11	−0.620	0.564	−0.169	−0.470
H12	−0.490	0.778	−0.193	−0.200
H13	−0.408	0.810	−0.220	−0.029
H14	−0.491	0.766	−0.347	−0.075
H15	−0.517	0.802	−0.256	−0.020
H16	−0.480	0.798	−0.265	0.035
H17	−0.474	0.612	−0.362	0.023
H18	−0.201	−0.486	−0.308	0.406
H19	0.384	−0.660	−0.204	0.265
H20	0.754	−0.419	0.215	0.088
H21	0.826	−0.315	0.263	0.063
H22	0.915	−0.255	0.275	0.045
H23	0.872	−0.225	0.377	0.072
H24	0.786	−0.160	0.510	0.092
Eigenvalue	6.852	6.551	5.314	1.009
CV %	0.286	0.558	0.780	0.822

**Table 3:** Results of the reliability analysis of the annual average hourly traffic volume ratios to AADT groups.

Hour Group	Early Morning Hours	Peak Hours	Day off-Peak Hours	Night Hours
Hours grouped	H01–H06	H07–H09 and H18–H19	H10–H17	H20–H24
Cronbach alpha value	0.8883	0.7314	0.9636	0.9596

**Table 4:** Scenarios for clustering analysis.

Scenarios	Clustering Variables
1	Early morning hours, peak hours, day off-peak hours, and night hours
2	Early morning hours, peak hours, and day off-peak hours
3	Early morning hours, peak hours, and night hours
4	Early morning hours, day off-peak hours, and night hours
5	Peak hours, day off-peak hours, and night hours
6	Early morning hours and peak hours
7	Early morning hours and day off-peak hours
8	Early morning hours and night hours
9	Peak hours and day off-peak hours
10	Peak hours and night hours
11	Day off-peak hours and night hours
12	Early morning hours
13	Peak hours
14	Day off-peak hours
15	Night hours

**Table 5:** Results of clustering analysis by scenario (in the order of the Hartingan–Wong value).

Scenarios	# of Variables	BetweenSS/TotSS			
		Hartigan–Wong	Lloyd	Forgy	MacQueen
14	1	0.850831	0.850831	0.838475	0.838475
15	1	0.841055	0.841055	0.841055	0.838978
12	1	0.836129	0.832705	0.832705	0.832705
7	2	0.810435	0.810435	0.771489	0.810435
8	2	0.805066	0.805066	0.798222	0.805418
11	2	0.788516	0.78794	0.78794	0.78794
4	3	0.771163	0.771163	0.771163	0.771163
13	1	0.770546	0.770415	0.770673	0.770415
6	2	0.704376	0.70012	0.704376	0.699581
9	2	0.692881	0.69014	0.692778	0.692674
2	3	0.638312	0.67237	0.672043	0.671204
5	3	0.611003	0.57226	0.624633	0.616671
1	4	0.608594	0.607309	0.614296	0.61716
10	2	0.579511	0.576184	0.577776	0.576366
3	3	0.570212	0.499304	0.565469	0.571325

**Table 6:** Result of fitness calculations.

Scenarios	Variables	Fitness (%)
8	Early morning hours and night hours	61.0
12	Early morning hours	61.0
15	Night hours	55.2
14	Day off-peak hours	53.3
7	Early morning hours and day off-peak hours	53.3

**Table 7:** Annual average hourly traffic volumes, annual average hourly traffic volume ratios to AADT, and ECDFs of each region.

Hour of Day	Urban			Suburban			Rural		
	AAHT	H	ECDF	AAHT	H	ECDF	AAHT	H	ECDF
1	308	0.0089	0.0089	156	0.0077	0.0077	48	0.0055	0.0055
2	204	0.0059	0.0148	98	0.0048	0.0125	30	0.0035	0.0090
3	158	0.0046	0.0193	76	0.0037	0.0162	24	0.0028	0.0118
4	158	0.0046	0.0239	78	0.0038	0.0201	26	0.0030	0.0148
5	260	0.0075	0.0314	130	0.0064	0.0265	44	0.0051	0.0198
6	620	0.0179	0.0493	338	0.0166	0.0431	102	0.0118	0.0316
7	1426	0.0411	0.0904	762	0.0375	0.0805	256	0.0295	0.0611
8	2388	0.0689	0.1593	1360	0.0669	0.1474	516	0.0595	0.1207
9	2520	0.0727	0.2320	1508	0.0742	0.2216	658	0.0759	0.1966
10	2078	0.0600	0.2920	1210	0.0595	0.2811	538	0.0621	0.2587
11	1982	0.0572	0.3491	1206	0.0593	0.3404	558	0.0644	0.3230
12	1912	0.0552	0.4043	1188	0.0584	0.3988	562	0.0648	0.3879
13	1812	0.0523	0.4566	1136	0.0559	0.4547	534	0.0616	0.4495
14	1954	0.0564	0.5130	1234	0.0607	0.5153	590	0.0681	0.5175
15	2030	0.0586	0.5715	1282	0.0630	0.5784	602	0.0695	0.5870
16	2080	0.0600	0.6315	1306	0.0642	0.6426	604	0.0697	0.6567
17	2200	0.0635	0.6950	1366	0.0672	0.7098	628	0.0725	0.7291
18	2546	0.0735	0.7684	1540	0.0757	0.7855	678	0.0782	0.8073
19	2506	0.0723	0.8407	1458	0.0717	0.8572	626	0.0722	0.8796
20	1760	0.0508	0.8915	954	0.0469	0.9041	358	0.0413	0.9209
21	1358	0.0392	0.9307	724	0.0356	0.9397	260	0.0300	0.9509
22	1092	0.0315	0.9622	566	0.0278	0.9675	202	0.0233	0.9742
23	794	0.0229	0.9851	402	0.0198	0.9873	138	0.0159	0.9901
24	516	0.0149	1	258	0.0127	1	86	0.0099	1

ACRONYMS: AAHT: annual average hourly traffic volume; H: annual average hourly traffic volume ratio to AADT; ECDF: empirical cumulative distribution function.

**Table 8:** Results of the two-sample goodness-of-fit tests (nationwide).

Test	Urban vs. Suburban			Suburban vs. Rural			Urban vs. Rural		
	KS	AD	CVM	KS	AD	CVM	KS	AD	CVM
Critical Stat.	0.01705	3.12×10^−7^	0.00190	0.02675	1.64×10^−6^	0.00499	0.03889	2.64×10^−6^	0.01286
*p*-value	0.00025	0.00025	0.00025	0.00025	0.00025	0.00025	0.00025	0.00025	0.00025

ACRONYMS: KS: Kolmogorov–Smirnov test; AD: Anderson–Darling test; CVM: Cramer–von Mises test.

**Table 9:** Results of the two-sample goodness-of-fit tests (principal level).

Division	Test	Urban vs. Suburban			Suburban vs. Rural			Urban vs. Rural		
		KS	AD	CVM	KS	AD	CVM	KS	AD	CVM
Gyeonggi-do	Crit. Stat.	0.01705	3.12×10^−7^	0.00190	0.02675	1.64×10^−6^	0.00499	0.03889	2.64×10^−6^	0.01286
	*p*-value	0.00025	0.00025	0.00025	0.00025	0.00025	0.00025	0.00025	0.00025	0.00025
Gangwon-do	Crit. Stat.	0.03352	1.40×10^−6^	0.00528	0.02163	6.69×10^−7^	0.00193	0.05438	0.00001	0.01341
	*p*-value	0.00025	0.00025	0.00025	0.00800	0.01300	0.02650	0.00025	0.00025	0.00025

ACRONYMS: KS: Kolmogorov–Smirnov test; AD: Anderson–Darling test; CVM: Cramer–von Mises test.

## Data Availability

Publicly available datasets were analyzed in this study. This data can be found here: http://www.road.re.kr/main/main.asp, accessed on 15 November 2021.

## References

[1] World Health Organization (WHO) Road Traffic Injuries. https://www.who.int/publications/i/item/9789241565684.

[2] Ministry of Land, Infrastructure and Transport (2021). 2021 National Traffic Safety Implementation Plan.

[3] American Association of State Highway and Transportation Officials (2010). Highway Safety Manual.

[4] Road Assessment Program and Its Application. https://english.koti.re.kr/user/bbs/BD_selectBbs.do?q_bbsCode=1073&q_bbscttSn=20191129145446743.

[5] The National Atlas of Korea. http://nationalatlas.ngii.go.kr/pages/page_562.php.

[6] May A.D. (1990). Traffic Flow Fundamentals.

[7] Albright D. (1987). A quick cluster control method: Permanent control station cluster analysis in average daily traffic calculations. Transp. Res. Rec..

[8] Flaherty J. (1993). Cluster Analysis of Arizona Automatic Traffic Record Data. Transp. Res. Rec..

[9] Zambon G., Benocci R., Bisceglie A., Roman H.E., Bellucci P. (2016). The Life Dynamap project: Towards a procedure for dynamic noise mapping in urban areas. Appl. Acoust..

[10] Transportation Research Board (2000). Highway Capacity Manual.

[11] Kang W. (2001). National Highway’s Design Criteria Based on Analysis of Functional Classification. J. Korean Soc. Transp..

[12] Kim J., Do M., Jeong J. (2002). Grouping method on functional classification for national highway. J. Korean Soc. Transp..

[13] Oh J., Lim S., Kim H. (2003). Classification of Road Type in According to the Traffic Characteristics. J. Korean Soc. Civ. Eng..

[14] Yu W., Jeong P. (2004). A Study on the Changes of the National Roads’ functional Classification by Traffic Volume Characteristics. J. Korea Plan. Assoc..

[15] Lim S., Oh J. (2005). A Study on Highway Classification and Traffic Characteristics by Highway Type. J. Korean Soc. Civ. Eng..

[16] Lim S., Oh J., Kim H. (2005). A Study on Discrimination and Traffic Characteristics of Recreational Roads. Seoul Stud..

[17] Cho J., Kim S., Rho J. (2008). A Study on Road Characteristic Classification using Exploratory Factor Analysis. J. Korean Soc. Transp..

[18] Cho J., Kim S. (2009). A Comparative Study on Statistical Clustering Methods and Kohonen Self-Organizing Maps for Highway Characteristic Classification of National Highway. J. Korean Soc. Civ. Eng..

[19] Choi B., Park C., Lee Y., Ki B., Lim T. (2010). Establishing criterion for classifying Urban/Rural freeway and suggestion of freeway operation strategies categorized by time and region. Yooshin Tech. Bull..

[20] Choi K., Won C., Chung W. (2007). Classification of Freeways based on the Characteristics of Hourly Traffic Variation for Efficient Network Planning. KSCE J. Civ. Eng..

[21] Korean Statistical Classification-Korean Administrative Divisions. http://kssc.kostat.go.kr/ksscNew_web/kssc/common/CommonBoardList.do?gubun=1&strCategoryNameCode=019&strBbsId=kascrr&categoryMenu=014.

[22] Local Autonomy Act. https://www.law.go.kr/lsSc.do?menuId=1&subMenuId=15&tabMenuId=81&eventGubun=060114#undefined.

[23] Ministry of Land, Infrastructure and Transport Regulations on the Road Structure and Facility Standards. https://www.law.go.kr/lsInfoP.do?lsiSeq=173203#0000.

[24] Ministry of Land, Infrastructure and Transport, The Korea Transport Institute (2017). 2017 National Traffic Survey, DB System Operation and Maintenance, and Traffic Analysis Network Establishment.

[25] United Nations Department of Ecomomic and Social Affairs (2018). World Urbanization Prospects, 2018 Revision.

[26] Devlieger I., Mayer A., Rosseel Y. (2016). Hypothesis testing using factor score regression. Educ. Psychol. Meas..

[27] Reio T.G., Shuck B. (2015). Exploratory Factor Analysis: Implications for Theory, Research, and Practice. Adv. Dev. Hum. Resour..

[28] Gorsuch R.L. (1983). Introduction. Factor Analysis.

[29] Smirnoff H. (1939). Sur les Ecarts de la Courbe de la Distribution Empirique. Receuil Math..

[30] Anderson T.W. (1962). On the distribution of the two-sample Cramer-von Mises criterion. Math. Statist..

[31] Lehmann E.L. (1951). Consistency and Unbiasedness of Certain Nonparametric Tests. Ann. Math. Statist..

[32] Anderson T.W., Darling D.A. (1952). Asymptotic theory of certain goodness-of-fit criteria based on stochastic processes. Ann. Math. Stat..

[33] Mann H.B., Whitney D.R. (1947). On a test of whether one of two random variables is stochastically larger than the other. Ann. Math. Stat..

[34] Kim S., Lee J. (2017). Power comparison of distribution-free two sample goodness-of-fit tests. Korean J. Appl. Statist..

[35] Engmann S., Cousineau D. (2011). Comparing distributions: The two-sample Anderson-Darling test as an alternative to the Kolmogorov-Smirnoff test. J. Appl. Quant. Methods.

[36] R Core Team (2021). R: The R Project for Statistical Computing.

[37] Road Act. https://www.law.go.kr/%EB%B2%95%EB%A0%B9/%EB%8F%84%EB%A1%9C%EB%B2%95.

[38] Ministry of Land, Infrastructure and Transport (2020). Road Work Manual.

[39] Ministry of Land, Infrastructure and Transport (2021). 2020 Statistical Yearbook of Traffic.

[40] Lee H. (2014). Research Methodology of Professor Hun Yeong Lee.

[41] Yu S. (2018). R-Statistical Analysis for Writing Academical Papers.

[42] Jia G., Zhou J. (2021). Effectiveness Evaluation Method of Application of Mobile Communication System Based on Factor Analysis. Sensors.

